# Evaluating the Beneficial Effect of Empagliflozin in Reducing Hospitalisation for Heart Failure in Patients With Type 2 Diabetes Mellitus: A Systematic Review

**DOI:** 10.7759/cureus.96593

**Published:** 2025-11-11

**Authors:** Akanksha Soni, Olabisi P Lawal, Japheth O Oyovwi, Happiness I Olaniyi, Wonderful O Anosike, Enibokun T Orobator, Nazeem Gabriels, Ibad Ali Khan Ghori, Aliyu O Olaniyi

**Affiliations:** 1 Acute Medical Unit, Stockport NHS Foundation Trust, Stockport, GBR; 2 Medical Laboratory Science, University of Benin, Benin City, NGA; 3 Geriatrics, Stepping Hill Hospital, Stockport, GBR; 4 Nursing, Barton Brook Care Home, Manchester, GBR; 5 General Adult Psychiatry, Cumbria, Northumberland, Tyne and Wear NHS Foundation Trust, Newcastle upon Tyne, GBR; 6 Global Health and Infectious Diseases, The University of Edinburgh, Edinburgh, GBR; 7 Emergency, Stockport NHS Foundation Trust, Stockport, GBR; 8 Internal Medicine, Stepping Hill hospital, Stockport, GBR

**Keywords:** cardiovascular risk, empagliflozin, heart failure, hospitalisation, hospitalization, randomised controlled trials, randomized controlled trials, sglt2 inhibitors, type 2 diabetes mellitus

## Abstract

Type 2 diabetes mellitus (T2DM) is associated with an increased risk of cardiovascular complications, with heart failure (HF) remaining a major cause of hospitalisation and mortality. Empagliflozin, a sodium-glucose cotransporter 2 (SGLT2) inhibitor, has demonstrated consistent cardioprotective effects in patients with type 2 diabetes mellitus (T2DM). Evidence shows that treatment with empagliflozin reduces the risk of hospitalisation for HF, with benefits that appear to extend beyond glucose lowering. These effects are thought to be mediated through mechanisms such as improved cardiac loading conditions, enhanced natriuresis, and optimised myocardial metabolism. Overall, empagliflozin represents an effective therapeutic option for reducing cardiovascular morbidity and hospital admissions in individuals with T2DM who are at risk for HF.

## Introduction and background

Type 2 Diabetes Mellitus (T2DM) is a major public health issue affecting about 537 million people all over the world, with scientific projections that about 643 million individuals will be affected by 2030, and 783 million by 2045 [1]. The leading cause of morbidity and mortality among T2DM patients is cardiovascular complications, and heart failure (HF) contributes largely to the mortality rate, although its effect is often under-recognised. Evidently, patients with T2DM have about two- to four-fold risk of having low ejection fractions, when compared with patients without diabetes [2,3]. Patients with T2DM have an increased risk of developing HF due to some shared pathophysiology, such as chronic inflammation, insulin resistance, poor glycemic control, and dysfunctional endothelium [4].

Sodium-glucose co-transporter-2 (SGLT2) inhibitors are a novel class of antihyperglycaemic agents which have a dual effect of controlling blood glucose and significant cardiovascular benefits. Prominent among all the SGLT2 inhibitors is empagliflozin, known for its ability to reduce hospitalisations related to HF (HHF) [5,6]. Moreover, the effect of empagliflozin on HHF has been associated with some actions, such as osmotic diuresis, improving myocardial contractility, and reducing preload and afterload [7,8].

One of the landmark studies that first demonstrated the benefits of empagliflozin on the cardiovascular system is the Empagliflozin Cardiovascular Outcome Event Trial in Type 2 Diabetes Mellitus Patients-Removing Excess Glucose (EMPA-REG OUTCOME) trial, which showed a significant reduction in hospitalisation for HF among patients with T2DM with established cardiovascular disease. Subsequent studies, such as EMpagliflozin outcomE tRial in Patients With chrOnic heaRt Failure With Reduced Ejection Fraction (EMPEROR-Reduced) and EMpagliflozin outcomE tRial in Patients With chrOnic heaRt Failure With Preserved Ejection Fraction (EMPEROR-Preserved) trials, substantiated the findings regarding empagliflozin by demonstrating the efficacy of the medications in patients with and without diabetes, with an established diagnosis of HF [9-11].

HHF is a significant event when considering the use of empagliflozin in patients with T2DM. Evidently, the risk of cardiovascular complications is higher in such patients, and HHF is a good predictor of long-term morbidity and mortality among them [12]. In view of this, a reduction in HHF is a meaningful and expedient outcome when evaluating the benefits of empagliflozin in T2DM with HF. Apparently, it is important to look for remedies that will reduce HHF with T2DM because prolonged hospitalisation and rehospitalisation are associated with a high mortality rate [13].

Despite evidence that empagliflozin provides cardiovascular benefits in patients with T2DM, there is a need to comprehensively evaluate its specific effect on HHF in this population. Previous studies often included heterogeneous patient populations or focused on composite cardiovascular outcomes, making it difficult to isolate the impact on HF alone. Therefore, it is important to examine the consistency and magnitude of empagliflozin’s benefit in patients with T2DM and HF and to determine whether treatment with empagliflozin can reduce the risk of HHF in these patients. Although there is some evidence that shows empagliflozin’s cardioprotective effects, few reviews have focused exclusively on patients with T2DM to evaluate the consistency and magnitude of its impact on HHF, thereby addressing a population-specific evidence gap.

Aim and objective

This review aimed to evaluate the effect of empagliflozin on the risk of HHF in patients with T2DM. The objectives were to identify and synthesize the available evidence on empagliflozin’s impact on HHF in such patients and to assess the consistency and strength of the reported clinical outcomes. It provides updated clinical insight into the consistency, magnitude, and translational relevance of empagliflozin’s importance in reducing HHF, by integrating findings from the latest randomised controlled trials (RCTs) and contextualizing outcomes within a diabetic population.

## Review

Methodology

This systematic review utilised the PICO framework, which is effective in formulating clinical research questions in evidence synthesis [14,15]. The PICO framework applied in this study was structured as follows: the Population (P) comprised adult subjects with T2DM; the Intervention (I) was empagliflozin; the Comparator (C) was placebo or standard diabetes care without SGLT2 inhibitors; and the Outcome (O) of interest was HHF, an important cardiovascular outcome in T2DM. Therefore, the research question guiding this review was: “Does Empagliflozin reduce the risk of HHF compared to placebo or standard care among adults with T2DM?”

Search Strategy and Database Selection

A comprehensive search was conducted in June 2025 in four leading electronic databases: Cumulative Index to Nursing and Allied Health Literature (CINAHL), EBSCO, Dimensions, and PubMed. These databases were chosen to optimise sensitivity because of their complementary coverage of clinical trials, clinical practice research, and biomedical literature. Search terms include terms for the disease condition (Type 2 diabetes mellitus), the intervention (empagliflozin) and the outcome measures (hospitalisation for heart failure).

The search term for PubMed was compiled as follows: (("Type 2 diabetes mellitus"[tiab] OR "Type 2 diabetes"[tiab] OR T2DM[tiab] OR "Non-insulin dependent diabetes"[tiab]) AND ("Empagliflozin"[tiab] OR "Jardiance"[tiab] OR "SGLT2 inhibitor"[tiab] OR "Sodium-glucose cotransporter 2 inhibitor"[tiab] OR "Sodium glucose transporter 2 inhibitor"[tiab] OR "Gliflozin"[tiab] OR "Gliflozins"[tiab]) AND ("Heart failure hospitalization"[tiab] OR "HF hospitalization"[tiab] OR "Heart failure events"[tiab] OR "HF events"[tiab] OR "Heart failure exacerbation"[tiab] OR "Decompensated heart failure"[tiab]))

Equivalent search terms were adapted for other databases (CINAHL, EBSCO, and Dimensions) using appropriate controlled vocabulary and Boolean operators. Additionally, reference lists of relevant systematic reviews were manually screened to identify any eligible studies not captured by electronic searches

Compiled search terms for other databases included (("Type 2 diabetes mellitus" OR "Type 2 diabetes" OR T2DM OR "Non-insulin dependent diabetes") AND ("Empagliflozin" OR "Jardiance" OR "SGLT2 inhibitor" OR "Sodium-glucose cotransporter 2 inhibitor" OR "Sodium glucose transporter 2 inhibitor" OR "Gliflozin" OR "Gliflozins") AND ("Heart failure hospitalization" OR "HF hospitalization" OR "Heart failure events" OR "HF events" OR "Heart failure exacerbation" OR "Decompensated heart failure"))

In addition to database searching, reference lists of relevant systematic reviews were hand-searched and included in the literature selection phase.

Eligibility Criteria

In order to make sure that the review includes strong and relevant evidence, criteria for inclusion and exclusion were predetermined. The included studies were limited to articles published in English language with published full text available and contain original findings involving participants with T2DM treated with empagliflozin. Among these publications, RCTs and observational studies (cohort and case-control studies) were included, provided they recorded HHF as an outcome.

Animal studies, studies involving children, or other SGLT2 inhibitors other than empagliflozin were excluded. Reviews, editorials, letters, case reports, and studies not presenting original data were also excluded. To ensure consistency in outcome, studies that did not provide data on the outcome variable (HHF) were also excluded. These eligibility criteria are consistent with criteria for targeted systematic reviews that aim to minimize bias and heterogeneity [16].

In this systematic review, where multiple publications arose from the same parent study, only the most comprehensive and relevant version was selected, ensuring that each dataset contributed uniquely to the synthesis. To ensure that duplication was avoided, only primary reports from distinct RCTs were included.

Study Selection and Data Extraction

All search results were imported into the EndNote reference manager (Clarivate, London, UK) to remove duplicates. The study selection process was conducted in two phases. In phase one, two reviewers independently screened titles and abstracts to identify potentially relevant studies. In phase two, full-text articles that met the inclusion criteria were retrieved and reviewed in detail. Any disagreements were resolved through discussion with a third reviewer to ensure objectivity and consistency.

A standardized data extraction form was used to collect key information from each eligible study, including author and publication year, study design, sample size, patient characteristics, number of HHF events, and reported effect estimates (hazard ratios or HRs with 95% confidence intervals or CI). Data were independently extracted and cross-checked by two reviewers, with discrepancies resolved through discussion with a third reviewer. 

Quality Assessment

The Revised Cochrane Risk-of-Bias tool for randomized trials (RoB 2) assessment tool was used to assess the methodological quality of the included studies [17]. This method ensured a systematic and standardized assessment of research quality [18]. Two reviewers conducted the assessment independently and resolved disagreements through discussion. 

*Data Synthesis* 

A narrative synthesis of the data extracted from the included studies was conducted to summarize study characteristics and key findings. The synthesis focused on comparing patient populations, study designs, interventions, and reported outcomes, particularly the effect of empagliflozin on HHF in patients with T2DM. Patterns and trends across the studies were highlighted to provide an overall assessment of the consistency and magnitude of the observed clinical benefits [19].

Literature Selection

A total of 1,133 articles were retrieved from five sources: 1,123 from electronic databases (155 from PubMed, 478 from EBSCO, 33 from CINAHL, and 457 from Dimensions) and 10 from reference lists. After applying the relevant filters, 920 articles were excluded; 892 during the screening process and 28 as duplicates-leaving 213 articles. Of these, 184 did not meet the inclusion criteria and were excluded, resulting in 29 articles eligible for full-text screening. Eight full-text articles were not retrievable, leaving 21 articles for full-text assessment. Following detailed evaluation, 15 articles were excluded for various reasons: five were meta-analyses, one did not report effect estimates, and nine did not provide data on HHF. Ultimately, six studies met the inclusion criteria and were included in this systematic review [5-7,9,11,20]. The study selection process is illustrated in the PRISMA flow diagram (Figure 1).

To ensure the inclusion of the most up-to-date evidence in this systematic review, we extended our literature search to June 2025, capturing recent trials published after 2022. This included pivotal studies such as Hernandez et al. (2024)[6] and Petrie et al. (2025) [7], which evaluated empagliflozin’s cardiovascular and heart failure outcomes in contemporary clinical settings. These newer studies provide additional insights into empagliflozin’s benefits beyond earlier landmark trials.

Characteristics of the included studies

Table 1 summarizes the key characteristics of the six RCTs included in this systematic review, each assessing the impact of empagliflozin on HHF among patients with T2DM.

**Table 1:** Key characteristics of the included studies P-values represent statistical comparisons between the empagliflozin and control groups. Statistical significance (p<0.05) reflects a lower incidence of heart failure hospitalisation in the empagliflozin group compared to the control.

Study ID	Study Design	Sample Size Empagliflozin (Empa) /Control	Experimental intervention	Comparator	Outcome assessed	Hospitalisation (Empa/Control)	Non-Hospitalisation (Empa/control)	Hazard Ratio (CI)	P-value
Fitchett et al. (2016) [20]	Randomised controlled Trial (RCT)	4687/2333	Empa (10 mg daily)	Placebo	Hospitalisation for heart failure (HHF)	126/95	4561/2238	0.65 (0.5-0.85)	0.002
Hernandez et al. (2024) [6]	Randomised, double-blind, placebo-controlled, event-driven trial	3260/3262	Empa (10 mg daily)	Placebo	HHF	148/207	3112/3055	0.67 (0.51-0.89)	0.006
Petrie et al. (2025) [7]	Randomised, double-blind, placebo-controlled trial (event-driven)	3260/3262	Empa (10 mg daily)	Placebo	HHF	148/207	3112/3055	0.67 (0.51-0.89)	<0.01
Zinman et al. (2015) [5]	RCT, double-blind, placebo-controlled	4687/2333	Empa (10 mg or 25 mg daily)	Placebo	Primary composite: Cardiovascular (CV) death, Non-fatal myocardial infarction (MI), or Non-fatal stroke	126/95	4561/2238	0.65 (0.5-0.85)	0.002
Packer et al. (2020) [9]	Randomised, double-blind, placebo-controlled, parallel-group, event-driven trial	1863/1867	Empa (10 mg daily)	Placebo	CV death or HHF	388/553	1475/1314	0.7 (0.58-0.85)	<0.001
Packer et al. (2021) [11]	Randomised, double-blind, placebo-controlled trial	1863/1867	Empa (10 mg daily)	Placebo	Composite of CV death, HHF, urgent HF visits	1364/1570	499/297	0.85 (0.75-0.95)	0.007

The studies conducted between 2015 and 2025 by Zinman et al., Fitchett et al., Packer et al., Hernandez et al., and Petrie et al. [5-7,9,11,20] were all randomized, double-blind, placebo-controlled trials. Sample sizes varied considerably, ranging from approximately 3,700 to over 7,000 participants. In each trial, the participants in the intervention arm received empagliflozin at a daily dose of 10 mg or 25 mg, while those in the control arm received a placebo in addition to standard diabetes care. The primary outcomes across studies included HHF, cardiovascular death, and composite endpoints involving cardiovascular events.

Among the six randomized controlled trials included in this study, Zinman et al. (2015) [5] and Fitchett et al. (2016) [20] represent the primary and secondary analyses of the EMPA-REG OUTCOME trial, respectively. These have therefore been treated as a single study in our synthesis to prevent data duplication and enhance clarity. To make this study more recent and to capture new evidence, more recent evidence from Hernandez et al. (2024) [6] and Petrie et al. (2025) [7] further strengthen the contemporary understanding of empagliflozin’s role in reducing HHFs.

Risk of bias scores for the included studies

Table 2 presents the quality assessment of the six included randomized controlled trials using the Revised Cochrane Risk-of-Bias Tool (RoB 2).

**Table 2:** Risk of bias score for all the included studies [5-7,9,11,20]

S/N	Study ID	Domain 1 (Bias from the randomisation process)	Domain 2 (Bias due to deviations from the intended interventions)	Domain 3 (Bias due to missing outcome data)	Domain 4 (Bias in measurement of the outcome)	Domain 5 (Bias in selection of the reported result)	Overall Assessment
1	Fitchett et al. (2016) [20]	Low	Low	Low	Low	Low	Low
2	Hernandez et al. (2024) [6]	Low	Low	Low	Low	Low	Low
3	Petrie et al. (2025) [7]	Low	Low	Low	Low	Low	Low
4	Zinman et al. (2015) [5]	Low	Low	Low	Low	Some concerns	Some concerns
5	Packer et al. (2020) [9]	Low	Low	Low	Low	Low	Low
6	Packer et al. (2021) [11]	Low	Low	Low	Low	Low	Low

Table 2 evaluates each study across five domains: bias from the randomization process (D1), bias due to deviations from intended interventions (D2), bias from missing outcome data (D3), bias in outcome measurement (D4), and bias in selection of the reported results (D5). All studies demonstrated a low risk of bias across most domains, reflecting strong methodological quality and internal validity. Only the study by Zinman et al. (2015) [5] showed “some concerns” under D5 regarding selective reporting, while all other trials, conducted by Fitchett et al. (2016), Hernandez et al. (2024), Packer et al. (2021) and Petrie et al. (2025) [6,7,9,11,20] were rated as low risk in all categories. Overall, Table 2 confirms that the included trials were of high quality, lending credibility and reliability to the review’s findings on the beneficial effects of empagliflozin in reducing HHF among patients with T2DM.

Results

A narrative synthesis of the six included RCTs was conducted to compare the effects of empagliflozin on HHF among patients with T2DM. Across all six studies, empagliflozin consistently demonstrated superior outcomes compared to placebo. The number of HHFs was notably lower in the empagliflozin groups, ranging from 126 to 388 events, compared with 95 to 553 events in the placebo groups. Reported HRs ranged from 0.65 (95% CI: 0.50-0.85) to 0.85 (95% CI: 0.75-0.95), all indicating statistically significant reductions in hospitalization risk (p<0.05). These consistent results across multiple large-scale RCTs highlight the robust cardioprotective effect of empagliflozin and support its role in reducing HHF among individuals with T2DM.

Discussion

This systematic review integrates evidence from landmark and recent clinical trials up to 2025. While earlier studies, such as the EMPA-REG OUTCOME trial (Zinman et al., 2015; Fitchett et al., 2016) [5,20] established the cardioprotective benefits of empagliflozin, newer investigations, including Hernandez et al. (2024) [6] and Petrie et al. (2025) [7] have expanded these findings to broader patient populations and reinforced the consistent reduction in HHFs.

Evidence from multiple high-quality RCTs indicates that empagliflozin reduces the risk of HHF in patients with T2DM compared to placebo or standard care. These findings are consistent across landmark cardiovascular outcome trials. For example, the EMPA-REG OUTCOME trial reported a 35% reduction in HHF events among patients taking empagliflozin [9,20]. Additional studies, including the EMPEROR-Reduced trial, support the efficacy of empagliflozin in lowering HF-related events, even in individuals without diabetes [21,22], highlighting its utility beyond glucose regulation.

The mechanisms through which empagliflozin provides cardiovascular protection extend beyond its glycaemic effects. These include osmotic diuresis, natriuresis, decreased arterial stiffness, enhanced cardiac energy efficiency, and attenuation of neurohormonal activation [4,23]. Collectively, these physiological effects may contribute to improved left ventricular function and reduced volume overload without stimulating the sympathetic nervous system [24,25], a major advantage in HF management.

Overall, the observed outcomes are consistent with the clinical guidelines from both the American Diabetes Association (ADA) and the European Society of Cardiology (ESC), which recommend the use of SGLT2 inhibitors in T2DM patients who either have established cardiovascular disease or are at elevated risk for heart failure [26]. The present findings offer additional justification for the early integration of empagliflozin into standard care for patients with T2DM, not only to optimize glycaemic control but also to prevent HF events and reduce hospitalization rates.

The mechanistic pathway that explains the cardioprotective effects of empagliflozin is another important aspect to be considered. Current evidence indicates that these benefits occur largely independent of glycaemic control, as demonstrated in trials including patients both with and without T2DM [27]. Empagliflozin enhances osmotic diuresis and increases renal sodium excretion, which causes a reduction in preload and afterload on the heart [28]. Apart from these haemodynamic effects, the drug shifts myocardial energy metabolism toward increased ketone-body utilisation, improving myocardial adenosine triphosphate (ATP) efficiency and function [29]. In addition to this, studies have shown that empagliflozin contributes to reversing cardiac remodeling by improving left ventricular ejection fraction and reducing ventricular volumes, thereby enhancing overall cardiac performance [30]. All these studies have provided strong evidence base that empagliflozin helps to reduce HHF. Moreover, empagliflozin reduces heart failure progression and rehospitalization by reducing the decline in estimated glomerular filtration rate (eGFR) and improving renal perfusion [27]

Evidently, the findings from this systematic review shift the paradigm: empagliflozin is increasingly recognized as a HF and cardiorenal therapy, rather than merely a glucose-lowering agent. It is important to state that early initiation in patients with T2DM at high cardiovascular risk could therefore prevent HF events and reduce the overall healthcare burden. It is recommended that future research should address some gaps, including long-term outcomes in real-world settings, sex-specific and ethnic differences in response, and cost-effectiveness analyses to guide global policy and practice [31,32].

Strengths and limitations

A key strength of this review lies in its inclusion of high-quality RCTs, which enhances the reliability and clinical relevance of the findings. The inclusion of well-structured clinical data provides a robust basis for evaluating the therapeutic value of empagliflozin in managing HF among individuals with T2DM.

Nevertheless, certain limitations must be considered. First, the limited number of included studies (n=6) may reduce the ability to assess variability through subgroup analyses. Although most studies were judged to be at low risk of bias, one trial raised some methodological concerns, which could influence overall interpretations. Moreover, inconsistencies in HF definitions, differences in patient demographics, and variable follow-up durations introduce the possibility of residual confounding.

The observed evidence should also be interpreted with caution regarding potential publication bias. Future studies should aim to include a larger number of trials, perform subgroup or stratified analyses, and further investigate sources of heterogeneity to refine understanding of empagliflozin’s effects across diverse patient populations.

## Conclusions

This systematic review of six high-quality RCTs involving over 25,000 participants found that empagliflozin significantly reduces the risk of HHF in patients with T2DM. The evidence consistently demonstrates the cardioprotective effects of empagliflozin beyond its glycaemia-lowering properties. Benefits were observed across diverse patient populations, supporting its role as an effective therapeutic option for patients with T2DM at risk of HF. Incorporation of empagliflozin into clinical practice may help reduce cardiovascular morbidity, hospital admissions, and associated healthcare costs, reinforcing current guideline recommendations for SGLT2 inhibitor use in high-risk individuals.
